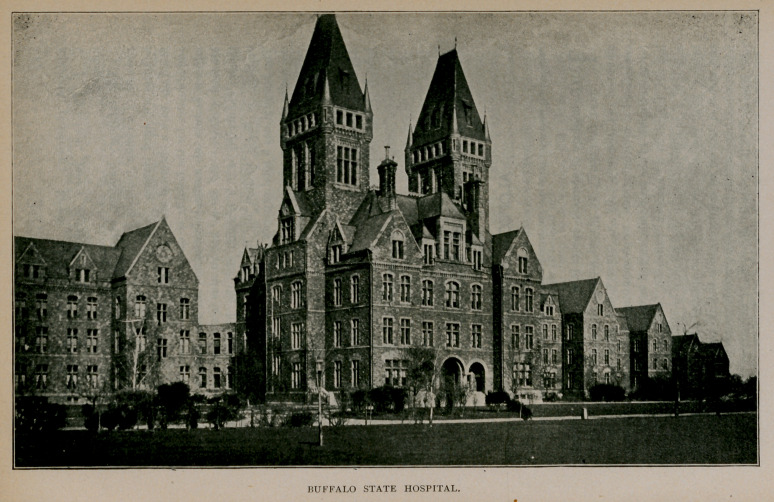# A Century of Medical History in the County of Erie.—1800-1900

**Published:** 1898-11

**Authors:** William Warren Potter

**Affiliations:** Buffalo, N. Y.


### A CENTURY OF MEDICAL HISTORY IN THE COUNTY OF ERIE.—1800–1900.

BY WILLIAM WARREN POTTER, M. D., Buffalo, N. Y.

*Pioneer Physicians—Medical Societies—Medical Colleges—Hospitals—Medical Journals—Medical Officers of the Civil War—Women Physicians—History of Homeopathy—Individual Members of the Profession.*

[*Continued from the October edition.*]

ALUMNI ASSOCIATION.—In 1871 the subject of organising an association of the alumni of Buffalo Medical College was agitated and many conferences on the subject were held between members of the faculty and prominent alumni. An organisation was finally perfected with Thomas D. Strong, '51, of Westfield, for its president. The first public meeting, however, was not held until February 23, 1875, and then in connection with the exercises of commencement day. An address to the alumni was delivered in the evening by Dr. William Warren Potter, in St. James's Hall, that stood on the site of the Iroquois Hotel. The graduating class was addressed on the same evening by Professor James P. White. At the first banquet of the association, held at the Tifft House, after the conclusion of the exercises at the hall, Dr. Thomas D. Strong presided, grace was said by the Rev. G. W. Heacock, D. D., and

Professor James P. White responded to the first toast, "Our Alma Mater."

Meetings have been held with regularity every year since 1875, large numbers of the alumni have attended each year and they have contributed to greatly increase the interest in commencement day. The part they have acted during the evening exercises and at the

GENERAL ALBERT J. MYER.

annual banquets has been a distinguishing feature of the ceremonies incident to the commencement exercises. Many of the alumni distinguished themselves during the civil war and some were found on almost every battle-field ministering to the wounded and otherwise performing duties as medical officers. Several of them received wounds while in the discharge of duty, and a number were taken prisoners. Some also have taken an active part in the recent war with Spain. It would be interesting to speak in detail of many who have so distinguished themselves, but a limited space prevents. It is fitting, however, that we should speak of one whose name and fame became coëxtensive with the boundaries of the globe. Albert J. Myer, of the class of '51, entered the United States Army as assistant surgeon in 1854, and soon afterward was assigned to duty in Texas. There he devised a single hand deaf-mute alphabet, and still later he invented and put into practical operation a system of military signals that was adopted by the army and which contributed inestimably to the success of our armies in the civil war.

A separate signal service bureau was created by act of congress, and Dr. Myer was placed at the head with the rank of Brigadier-general. After the war General Myer, who was gifted with a scientific mind as well as with inventive genius, prepared a code of weather signals that has become the basis of the present system in operation throughout the world, and which gained for him the familiar title of "Old Probabilities." He died at Buffalo, August, 1880, and his remains rest in a beautiful mausoleum in Forest Lawn Cemetery.

### Medical Department, Niagara University.

In 1856 an academic school, called the Seminary of Our Lady of Angels, was established near Suspension Bridge. In 1883 this seminary was erected into a university by the regents at Albany, with authority to establish any of its colleges in Erie county. A department of medicine was thereupon organised by Niagara University, which was located in the City of Buffalo. The chief promoter of the enterprise was the late Dr. John Cronyn, of Buffalo, who in connection with Bishop Ryan and the other officers of the university succeeded in establishing the new medical school in season to begin operations in September, 1883. Its requirements for admission, instruction and graduation were that students must pass a matriculation examination in such branches as were considered necessary to fit them for the study of medicine; a course of three years' study to comprise three full lecture terms of six months each; and a final examination by a separate board appointed by the trustees. These demands were in advance of the requirements then usually in force.

The first faculty consisted of the following-named gentlemen: John Cronyn, professor of the principles and practice of medicine and clinical medicine, president; Thomas Lothrop, professor of obstetrics, vice-president; William H. Heath, professor of descriptive and surgical anatomy; Augustus R. Davidson, professor of chemistry, pharmacy and toxicology; Henry D. Ingraham, professor of gynecology and diseases of children; Charles G. Stockton, professor of materia medica and therapeutics; Charles C. F. Gay, professor of operative surgery; William S. Tremaine, professor of the principles and practice of surgery and clinical surgery; Clayton

MEDICAL DEPARTMENT, NIAGARA UNIVERSITY.

M. Daniels, professor of clinical surgery, physiology and microscopy; Alvin A. Hubbell, professor of ophthalmology, otology and laryngology; Hon. J. M. Congdon, professor of jurisprudence. The Rt. Rev. Stephen V. Ryan, D. D., was announced as chancellor of the university and John L. C. Cronyn was appointed demonstrator of anatomy. Of these Drs. Lothrop, Ingraham and Hubbell now hold professorships in Buffalo University Medical College. Bishop Ryan, Drs. Cronyn, Davidson, Tremaine and Gay are dead; Drs. Stockton and Heath are teaching in Buffalo University; Drs. Daniels, Fell, John L. C. Cronyn and J. M. Congdon resigned.

Dr. C. C. F. Gay, who first occupied the chair of surgery, was distinguished in his department. He served on the surgical staff of the General Hospital as well as that of the Sisters of Charity Hospital, and withal was an eminent citizen. His death occurred March 27, 1886. In 1889 Dr. Herman Mynter was appointed to the chair of surgery, which he continued to hold until the college was merged with that of Buffalo University in June, 1898.

The college opened with a class of ten students. The first lectures were delivered at the Buffalo Hospital of the Sisters of Charity, and later, lecture rooms were secured in the Young Men's Christian Association building. In 1884 a college edifice, located on Ellicott street, between Broadway and Clinton, was constructed and made ready for occupation about January 1, 1885. In 1891 this building was enlarged to greater proportions to meet the increased demands for larger laboratories and ampler lecture rooms.

Dr. Augustus R. Davidson, who was at first professor of chemistry and to whose chair later the department of dermatology was added, died May 25, 1888, aged 43 years. His death was a severe blow to the college and the vacancy created thereby was not easily filled. The chair of chemistry was divided, William H. Pitt becoming professor of general chemistry and physics and John A. Miller, professor of medical chemistry and toxicology.

The first commencement exercises of the college were held at Association Hall on the evening of April 12, 1886, at which time the degree of doctor of medicine was conferred upon the following-named candidates: Edward J. Murphy, Thomas Hill, George W. T. Lewis and Anthony Hill, Buffalo; Ravell B. Parks, Jamestown; George M. Wetherill, Toronto. The method of conferring degrees adopted by this college was known as "hooding," an ancient rite observed in many English universities.

The first address to the graduates was delivered by Dr. Simeon T. Clark, of Lockport, professor of medical jurisprudence, who had been appointed to the chair, vice Joseph M. Congdon, resigned. Dr. Clark, a gifted and versatile man, was seized with apoplexy while in the performance of his professional duties, and died in the midst of a useful life, December 24, 1891.

As before stated, this college, in obedience to the growing sentiment in favor of consolidating medical schools wherever practicable, was united with the Medical Department of the University of Buffalo, June, 1898. It, however, has left the influence of its good work upon the profession of this region and will always be respected for the stand it took in favor of higher medical education at a time when sacrifices were required to establish a school on the lines it adopted.

Alumni Association.—An alumni association of Niagara University was organised in 1886, consisting of the faculty and lecturers of the college, together with the graduates of that year. The officers were as follows: President, William H. Heath, Buffalo; first vice-president, R. B. Parks, Jamestown; second vice-president, E. J. Murphy, Buffalo; secretary, George W. T. Lewis, Buffalo; treasurer, Simeon T. Clark, Lockport. Executive committee, F. S. Crego, S. T. Clark, Anthony Hill, Buffalo.

The first public meeting of the alumni association was held April 12, 1887, at which Dr. William H. Heath presided. Papers were read at this meeting by Drs. Stephen Smith, of New York; B. H. Daggett, H. D. Ingraham and Frank H. Potter, of Buffalo. The first banquet was held at the Genesee Hotel, in which the faculty, alumni and invited guests participated. The association continued to hold its annual meetings at the college hall on the commencement day of the medical school during the life-time of the college.

When this college was organised, two years' study in medicine was among the legal requirements, but Niagara University established a three years' curriculum, recommending, however, a four years' course. The law requiring the medical schools of the State of New York to establish three years as the minimum course of medical study took effect September 1, 1891, and by an amendment passed March 21, 1896, a four years' course of collegiate study was established as the minimum requirement in this state, that took effect January 1, 1898.

### III.—Hospitals.

### Buffalo Hospital of the Sisters of Charity.

Though it had been many times before proposed to establish a hospital in Buffalo, plans did not materialise until 1848, when the first hospital in the city was really opened for the reception of patients. A building located on what is now known as Pearl Place, made up of a group of several contiguous dwelling-houses that had been occupied

BUFFALO HOSPITAL OF THE SISTERS OF CHARITY—1848–1876.

previously as an orphan asylum, now (1898) used as a tenement, was converted into a hospital and placed under the management of the Sisters of Charity. It was incorporated under the laws of the State of New York, and accommodations were provided for 100 patients. Later an appropriation of $9,000 was made by the state. It was pro vided that no questions should be asked of patients when admitted touching matters of religion, and that applications for admission should be made to the medical board, the president of the Good Samaritan Society, and to the Society of St. Vincent of Paul; and further that a line from the pastor of any church of whatsoever denomination should also secure admittance by patients for treatment.

The building was made ready in 1848, and during the first six months 121 patients were received. The first medical board was constituted as follows: Frank Hastings Hamilton, attending surgeon; Austin Flint, attending physician, and Josiah Trowbridge, consulting physician. Appreciating the importance of clinical instruction, the late Bishop Timon, a learned prelate of the Roman Catholic church, threw open the doors of the hospital for that purpose, and for a small fee the students of the medical college then, lately established, received bedside training under the supervision of an attending physician or surgeon.

During the cholera epidemic of 1849 there were admitted into the institution previous to September 1st, 136 patients suffering from this disease, 52 of whom died. The report of the hospital for the year 1849, issued November 27th, shows that 1,513 patients in all were admitted, of whom more than one-half were charity beneficiaries.

From time to time the capacity of the hospital was increased, so that finally it aggregated accommodation for 200 patients. At the end of twenty-five years, however, it had outgrown the limits of its first location, and in 1872 a site was purchased on North Main Street, corner of Delevan Avenue, on which it was proposed to build a new and larger hospital. In June, 1875, ground was broken, in August the corner-stone was laid, and on November 5, 1876, the hospital was dedicated. The cost of the building and ground was $168,368. The building is a large, substantial, four-story brick structure with basement, situated upon high ground and surrounded by broad lawns. A new wing has lately been constructed, and the hospital as it now stands is a comely modern building with all the conveniences necessary for its numerous patients. It has its own electric plant for lighting, and is heated and ventilated according to the latest and best methods. It has large and well-appointed surgical and gyneco-

BUFFALO HOSPITAL OF THE SISTERS OF CHARITY—1898.

logical operating rooms, both of which are especially complete in modern equipments. The total cost of the building as it now stands has been about $250,000, and it has a capacity of 334 beds. A contagion pavilion has also been erected containing twenty-five or thirty beds. This was one of the first hospitals in the United States under the management of the Sisters of Charity to establish the custom of resident physicians, and it was likewise the first under the Sisters' management to establish a training school for nurses.

### BUFFALO GENERAL HOSPITAL.

Meetings of several prominent citizens were held at the office of Henry W. Rogers, collector of the port of Buffalo, on the 23d and

BUFFALO GENERAL HOSPITAL—1858.

26th days of October, 1846, at which an association was formed for the establishment of a public hospital. Thirty-five directors were appointed and officers were elected as follows: President, Josiah Trowbridge; first vice-president, General Heman B. Potter; second vice-president, George W. Clinton; secretary, E. S. Baldwin; treasurer, S. N. Calender. Executive committee: R. H. Haywood, Bryant Burwell and George Jones. Dr. Frank Hastings Hamilton was appointed attending surgeon, Dr. Austin Flint, attending physician, and Drs. Trowbridge and Burwell respectively, consulting physician and consulting surgeon.

It was soon announced that the building known as the Seaman's Home had been obtained for temporary use as a city hospital, but before the plan fully developed the organisation seemed to have collapsed. Opposition was met with and an appropriation which had been nearly obtained from the state was lost. Though the necessity for a hospital was great, yet the next year the Buffalo Hos-

BUFFALO GENERAL HOSPITAL AS ENLARGED—1880.

pital of the Sisters of Charity went into operation and this met the existing emergency.

The rapid growth of the city, however, soon created the necessity for another hospital; hence in 1854 a second attempt to establish one was made. A board of fifty trustees was created with Millard Fillmore at its head. It was thought inadvisable to commence operations without a capital of $100,000, and as the money could not be raised, this project, too, was abandoned. A little later, however, a board of nine trustees was appointed, consisting of Charles Clark, president; Andrew J. Rich, vice-president; William T. Wardwell, secretary and treasurer; George S. Hazard, Bronson C. Rumsey, Roswell L. Burrows, Stephen C. Howell and Henry Martin. On the 21st of November the association was formed and the certificate of incorporation was filed in the County Clerk's Office December 13, 1855. The sum of $20,000 was subscribed by citizens and in 1857 the hospital received an appropriation from the state of $10,000 more, which, together with the first sum, created a fund sufficient to enable the association to begin operations.

A building was erected on High Street on a site that was considered one of the finest in the city, having 361 feet front on High, 450 on Goodrich and a depth of 282 feet. The west wing of the building was rapidly pushed to completion and was dedicated June 26, 1858, with appropriate ceremonies amidst an enthusiastic gathering of citizens, and on which occasion an address was delivered by the Hon. James O. Putnam, that was full of patriotism, charity and lofty eloquence.

The following-named physicians were appointed medical officers for one year dating from July 1, 1858: Attending physicians, James M. Newman, Thomas F. Rochester and C. C. Wyckoff; consulting physicians, James P. White, George N. Burwell and P. H. Strong; attending surgeons, Charles H. Wilcox, Sandford Eastman and Austin Flint, Jr.; consulting surgeons, Frank Hastings Hamilton, C. C. F. Gay and John Root. Dr. Walter B. Coventry was the first resident physician. A new wing was afterward erected, that was dedicated October 1, 1880, bringing the capacity of the hospital up to 150 beds, and soon after a training school for nurses was instituted that has been in successful operation ever since it was established. A nurses' home has been built upon the hospital grounds.

The demands made by the large increase in growth of the city were such as to overflow the capacity of the hospital and a further enlargement was therefore determined upon, which is now in process of building. Through the munificent gift of $55,000, made by Mrs. George B. Gates and her three daughters, Mrs. William Hamlin, Mrs. Charles W. Pardee and Miss Elizabeth Gates, it was rendered possible to begin this work last year (1896) and it is now in process of construction. When completed it will be one of the most substantial and beautiful hospital structures in the country. The

PROPOSED BUFFALO GENERAL HOSPITAL.

(From The Illustrated Buffalo Express.) (Copyright, 1898, by George E. Matthews & Co.)

hospital staff is largely made up of the faculty of the Buffalo University Medical College, though Dr. C. C. Wyckoff and Dr. Conrad Diehl are still consulting physicians.

### Buffalo State Hospital.

Commissioners were appointed by Gov. John T. Hoffman in 1869 to locate a hospital in Western New York to be devoted to the care and treatment of the insane. The names of these commissioners were as follows: Dr. John P. Gray, Utica; Dr. James P. White, Buffalo; Dr. Thomas D. Strong, Westfield; Dr. William B. Gould, Lockport, and Dr. Milan Baker, Warsaw. After a number of meetings and the examination of several proposed localities it was finally determined to establish the hospital at Buffalo, and it is appropriate to state in this connection that it was chiefly due to the efforts of Dr. James P. White and Mr. Joseph Warren that the hospital was located here.

The corner-stone of the institution was laid September 18, 1872, with masonic rites, in the presence of a large number of citizens. Governor Hoffman was present and took part in the ceremonies in an appropriate speech. Dr. James P. White, president of the board of managers, made some introductory remarks, and the Hon. James O. Putnam delivered a formal address. The first board of managers was made up as follows: Dr. John P. Gray, Utica; Asher P. Nichols, Dr. James P. White, William G. Fargo, Joseph Warren and George R. Yaw, of Buffalo, Dr. William B. Gould, Lockport; Lorenzo Morris, Fredonia, and Augustus Frank, Warsaw.

The erection of the administration building and the east wing was proceeded with at once, but it was not until 1880 that the hospital was made ready for the reception of patients. Dr. Judson B. Andrews, the first assistant physician at the state hospital at Utica, was appointed superintendent, and under his able management the hospital soon assumed a leading position among institutions for the care of the insane in this country. Work on the west wing began in 1889, and the first building was completed in 1891; the second building in 1895, and the three remaining buildings are now ready for occupancy. It is one of the most ornamental, extensive and substantial structures of the kind perhaps in the world. A training school for nurses was established in 1886, this being the first public institution for the insane to establish such a school in this country. Over 100 graduates have been sent out, many of whom are doing private nursing throughout the United States. The hospital also has a nurses' home erected upon the grounds.

The medical staff is at present made up as follows: Arthur W.

JUDSON B. ANDREWS, M. D.—1834–1894.

Hurd, Superintendent; Henry P. Frost, first assistant; George G. Armstrong, second assistant; Walter H. Conley, assistant physician; Helene Kuhlmann, woman physician; Joseph B. Betts,

BUFFALO STATE HOSPITAL.

assistant physician; Edwin A. Bowerman, junior physician; C. J. Patterson, junior physician; Edward G. Aldrich, medical interne.

The infirmary building, begun last year, is now completed, and consists of a center building for acute cases, with two wings for the helpless and aged classes. The central building is very completely fitted up with a chemical and physiological laboratory and a large amphitheater for the holding of clinics in mental diseases, which is a regular feature of medical instruction in the Buffalo University Medical College. So far as known this is the first clinical amphitheater connected with a hospital for the insane. The infirmary building is finely located on Elmwood avenue, facing the Park. The hospital has a total capacity of 1,631, and the aggregate cost of the entire structure was over two millions of dollars.

The following-named persons compose the present board of managers: Joseph P. Dudley, president; Daniel H. McMillan, vice-president; Thomas Lothrop, M. D., Frederick P. Hall, James Atwater, Jessie Holland Jewett and Esther K. McWilliams, with John E. Pound, attorney.

(*Continued next month.*)

## Figures and Tables

**Figure f1:**
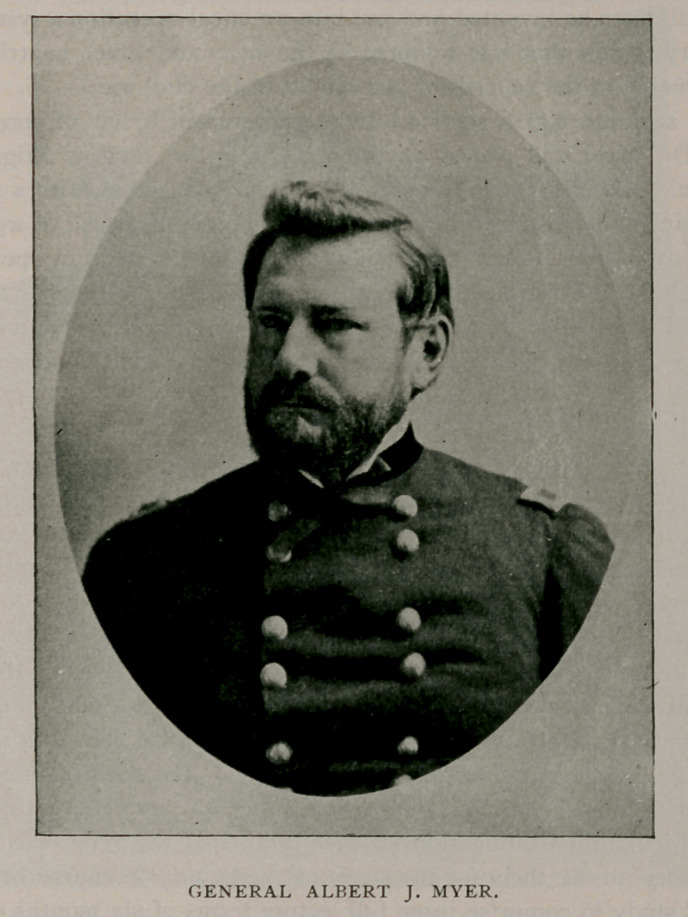


**Figure f2:**
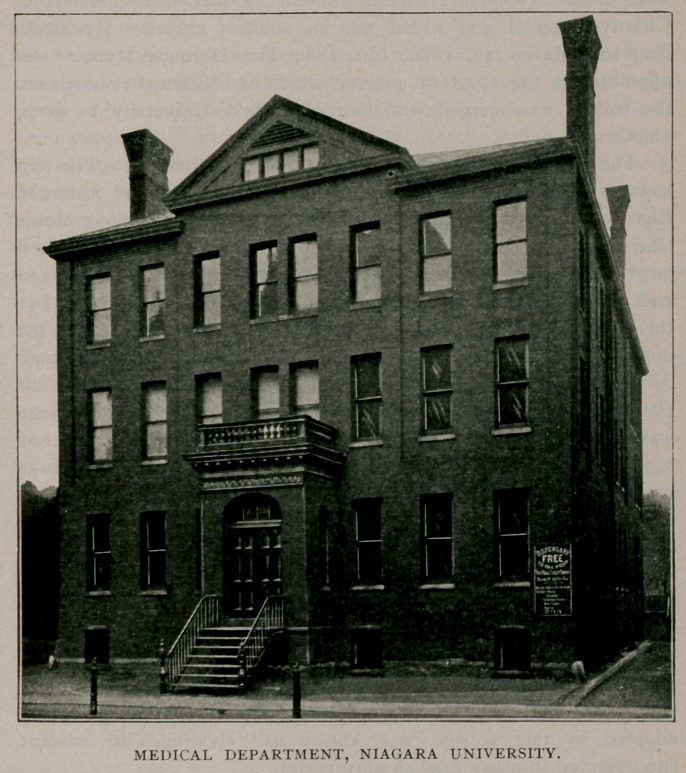


**Figure f3:**
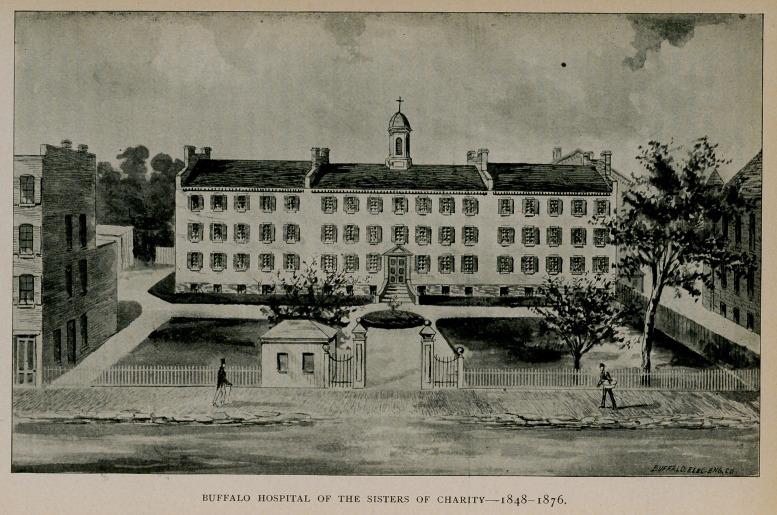


**Figure f4:**
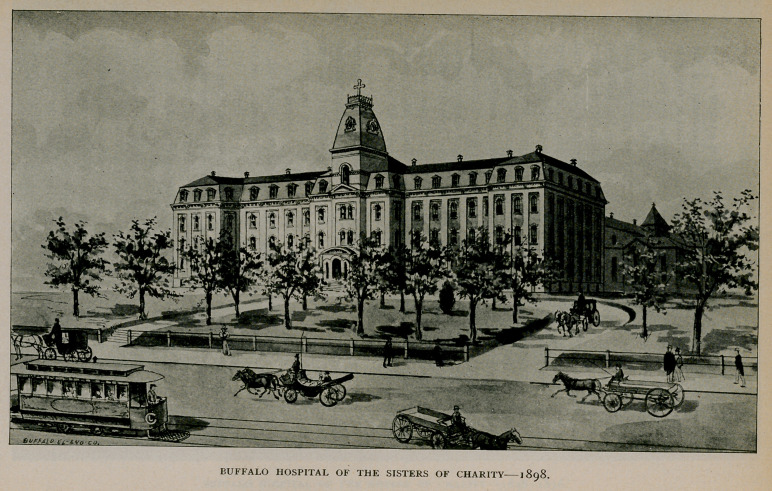


**Figure f5:**
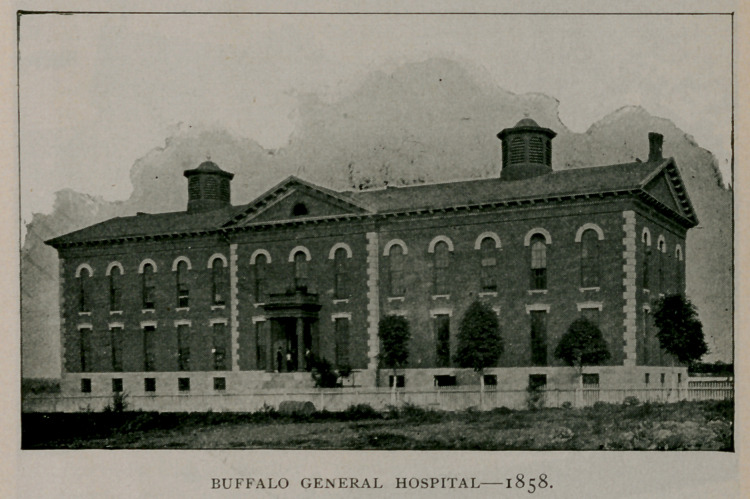


**Figure f6:**
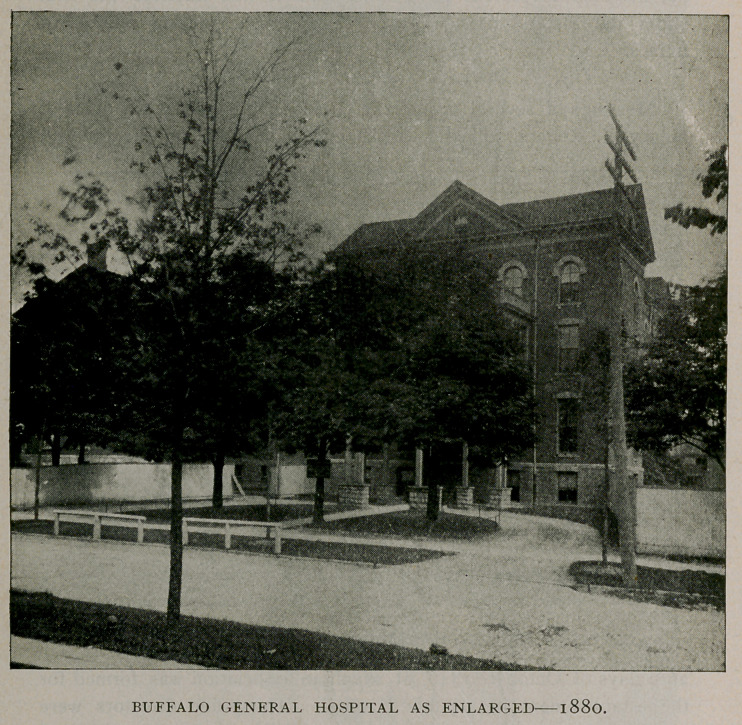


**Figure f7:**
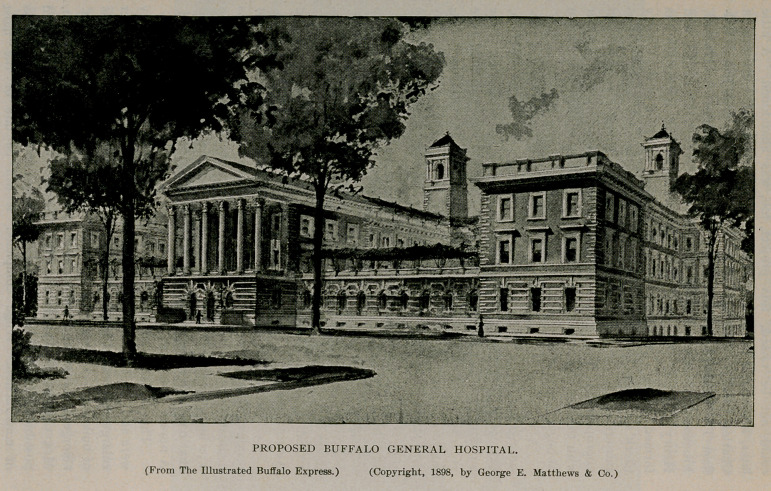


**Figure f8:**
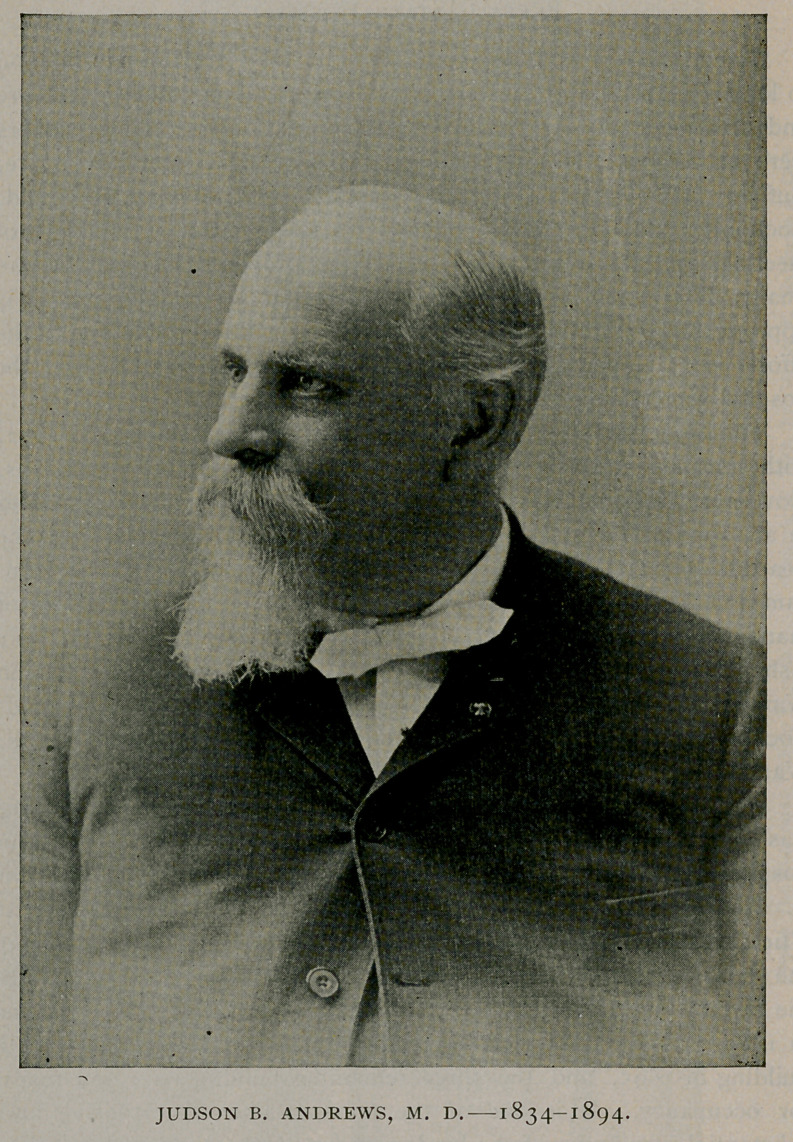


**Figure f9:**